# The Development of a High-Strength Mg-10.3Gd-4.4Y-0.9Zn-0.7Mn Alloy Subjected to Large Differential-Thermal Extrusion and Isothermal Aging

**DOI:** 10.3390/ma16186103

**Published:** 2023-09-07

**Authors:** Kui Wang, Xinwei Wang, Jinxing Wang, Cong Dang, Xiaoxu Dou, Song Huang, Manping Liu, Jingfeng Wang

**Affiliations:** 1 School of Material Science and Engineering, Jiangsu University, Zhenjiang 212013, China; 2 National Engineering Research Center for Magnesium Alloys, College of Materials Science and Engineering, Chongqing University, Chongqing 400044, China; 3 Chongqing Special Equipment Inspection and Research Institute, Chongqing 401121, China

**Keywords:** magnesium alloy, extrusion, large differential-thermal extrusion, microstructure, mechanical properties

## Abstract

The large differential-thermal extrusion (LDTE) process, a novel approach for efficiently fabricating a high-strength Mg-10.3Gd-4.4Y-0.9Zn-0.7Mn (wt.%) alloy, is introduced in this work. Unlike typical isothermal extrusion processes, where the ingot and die temperatures are kept the same, LDTE involves significantly higher ingot temperatures (~120 °C) compared to the die temperature. For high-strength Mg-RE alloys, the maximum isothermal extrusion ram speed is normally limited to 1 mm/s. This research uses the LDTE process to significantly increase the ram speed to 2.0 mm/s. The LPTE-processed alloy possesses a phase composition that is similar to that of isothermal extruded alloys, including α-Mg, 14H-type long-period stacking ordered (LPSO) and β-Mg_5_(Gd, Y) phases. The weakly preferentially oriented α-Mg grains in the LDTE-processed alloy have $<10\bar{1}0>$_Mg_//ED fibrous and <0001>_Mg_//ED anomalous textures as their two main constituents. After isothermal aging, high quantitative densities of prismatic β′ and basal γ′ precipitates are produced, which have the beneficial effect of precipitation hardening. With a yield tensile strength of 344 MPa, an ultimate tensile strength of 488 MPa, and an elongation of 9.7%, the alloy produced by the LDTE process exhibits an exceptional strength–ductility balance, further demonstrating the potential of this method for efficiently producing high-strength Mg alloys.

## 1. Introduction

Magnesium alloys have garnered growing attention in recent years as a viable structural material for transportation, aircraft, and electronic devices owing to their high specific strength, low density, and recyclability [1,2,3], whereas the limited strength and poor formability of Mg alloys have constrained their pervasive usage, especially in the load-bearing components of lightweight structures [4,5,6]. Recent research efforts have been focused on developing high-strength Mg alloys with enhanced formability and ductility through introducing rare-earth (RE) and other alloying elements. Mg-Gd-Y-Zn alloys, in particular, outperform most commercial magnesium alloys in terms of their mechanical properties because of the formation of RE-rich intermetallic phases, including long-period stacking-ordered (LPSO), basal (γ″ and γ′), and prismatic (β″, β′ and β_1_) phases [7,8,9]. Although these intermetallic phases are crucial for strengthening alloys and lowering recrystallization temperatures during deformation processing, some of them and their maternal phases also significantly deteriorate the alloy’s forming properties, significantly increasing the deformation resistance, and narrowing the effective window for plastic processing.

Extrusion is a widely used process for producing Mg alloys because it refines the grain size, homogenizes the microstructure, and regulates the texture [10,11,12]. High-strength Mg-Gd-Y-Zn alloys are frequently produced using traditional isothermal extrusion methods at slow extrusion speeds, where the extrusion ram does not exceed 1 mm/s [13,14,15,16]. This approach helps to prevent the overburn of intermetallic phases, ensuring a good surface quality, tight dimensional accuracy, and the absence of defects during processing [10]. C. Xu et al. [17] successfully isothermally extruded a high-strength Mg-based alloy (Mg-7.5Gd-2.5Y-3.5Zn-0.9Ca-0.4Zr, wt.%) that exhibited favorable mechanical properties. The alloy achieved an ultimate tensile strength (UTS) of 405 MPa, yield tensile strength (TYS) of 345 MPa, and an elongation (EL) of 8.8% when extruded with 0.1 mm/s ram speed. Analogously, a high-strength Mg-9.5Gd-4Y-2.2Zn-0.5Zr alloy was developed through isothermal extrusion with a 0.1 mm/s ram speed [15]. The resultant alloy exhibited a high ratio of dynamically recrystallized grains, along with densely dispersed fine precipitates, leading to improved strain accommodation and reduced early fracture without sacrificing work hardening. This resulted in an optimized mechanical performance, with a UTS of 535 MPa, TYS of 461 MPa, and elongation of 6.1%. Nevertheless, slow extrusion rates can be costly and time-consuming, limiting the practical use of high-strength Mg alloys.

The dynamic precipitation of Gd/Y-enriched phases, determined by the temperature-sensitive solid solubility of Gd and Y, is known to significantly impact the mechanical behavior of extruded Mg alloys [18,19,20]. Low-extrusion die preheating temperatures facilitate rapid cooling and the significant retention of solid-solution elements in the magnesium matrix, which may aid the age-hardening process. High ingot preheating temperatures, on the other hand, result in a higher concentration of solid-solution elements, which may also contribute to an improved aging hardening effect [21]. According to this idea, a differential-thermal extrusion procedure was proposed that makes use of a significant temperature difference between the ingot and extrusion die to promote microstructure refinement, stimulate aging precipitation, and improve mechanical properties [22,23,24]. Recently, a Mg-15Gd-1Zn-0.4Zr alloy was produced utilizing a differential-thermal extrusion process followed by aging [24]. At a 0.2 mm/s ram speed, the resultant material was subjected to tensile testing and exhibited a UTS of 461 MPa, TYS of 380 MPa, and elongation of 2.7%. It is foreseeable that high ingot temperatures during extrusion soften alloys, and low-extrusion die temperatures result in restricted temperature increases. Therefore, further investigations into the influence of extrusion speed on high-strength Mg-Gd-Y-Zn alloy microstructures and mechanical properties employing a differential-thermal extrusion technique should be worthwhile.

In this study, we seek to better understand how the microstructure and mechanical properties of the alloy Mg-10.3Gd-4.4Y-0.9Zn-0.7Mn (VWZM10411) are affected by large differential-thermal extrusion (LDTE). The LDTE process is conducted at a temperature difference of more than 100 °C between the ingot and die, resulting in an improved extrusion speed and optimized mechanical properties. The semi-continuous casting VWZM10411 alloys are subjected to LDTE and isothermal aging, and the microstructure and mechanical properties are characterized in detail. The results of this study provide valuable insights into the mechanisms by which the LDTE process affects the microstructure and mechanical properties of Mg-Gd-Y-Zn alloys and contribute to the advancement of high-strength Mg alloys.

## 2. Experimental Procedures

Starting metals utilized in this study were 87 mm-diameter Mg-10.3Gd-4.4Y-0.9Zn-0.7Mn alloy ingots produced by semi-continuous casting. A multi-stage heat treatment was employed to homogenize the as-cast alloy ingots, consisting of sequential heating at 450 °C for 8 h, 500 °C for 8 h, and 525 °C for 5 h, followed by quenching in 90 °C warm water. Cylindrical samples with 82 mm diameters were produced mechanically from the homogenized alloy ingots.

For high-strength Mg-Gd-Y-Zn alloys with an extrusion ratio of 8–20, it was crucial to operate within a RAM speed range of 0.3 to 2 mm/s. This choice was informed by previous research and experimental findings [13,14,15,16,17], which showed that RAM speeds exceeding 2 mm/s can result in adverse outcomes, such as incomplete material structure formation or even material structure overburning. Conversely, RAM speeds below 0.3 mm/s may result in high resistance to deformation or non-uniform deformation, making extrusion difficult or even impossible.

**Figure 1:**
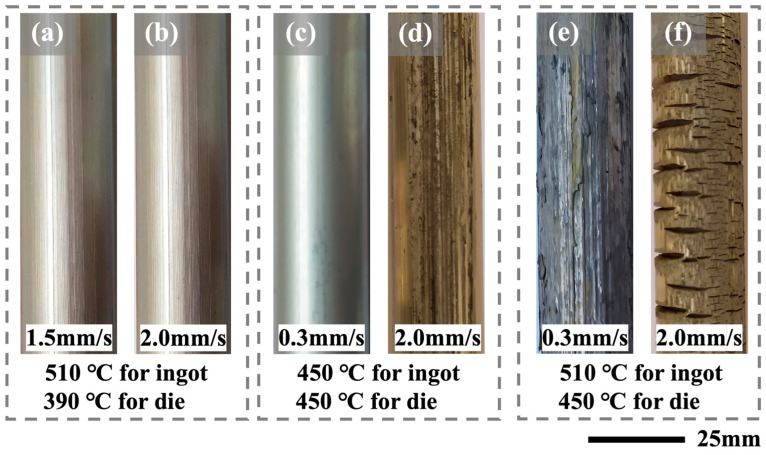
Photographic images showing the surface quality of the VWZM10411 alloy rods extruded at various extrusion processes: (**a**–**f**) for E1–6 extrusion rods.

The extrusion process was conducted using a 500 ton industrial extruder. To begin, the homogenized ingots underwent preheating at two distinct temperatures: 450 and 510 °C, each lasting for 30 min. In addition, the extrusion dies were preheated to different temperatures, specifically 390 and 450 °C, prior to the extrusion operation. Then, the extrusion was performed with a fixed extrusion ratio of 11:1 and at various ram speeds, namely, 0.3, 1.5, and 2.0 mm/s, respectively. Detailed extrusion conditions are summarized in Table 1. Extruded rods were cooled under ambient conditions and had a diameter of 25 mm, as shown in Figure 1. The surface quality of the rods E1 to E3 was exceptional, with a lustrous metallic appearance and no visible macroscopic defects. The E4 and E5 rods, on the other hand, were severely oxidized and showed signs of overburning. Periodic cracks of varying sizes were discovered in the E6 rod. Compared with the isothermal extruded E3 alloy rod, the E2 alloy rod produced using the LDTE method demonstrated a faster extrusion speed while maintaining comparable surface quality. As a result, rather than on the E4–6 rods, the detailed analysis and discussion in the following sections concentrate on the E2 and E3 alloy rods. The E2 sample was subjected to an interrupted extrusion experiment to investigate microstructure evolution. The extruded samples were then subjected to a 50 h isothermal aging process at 200 °C.

X-ray diffraction (XRD, Rigaku D/Max2500PC, Tokyo, Japan) with a copper target was used to investigate the phase identification of the extruded alloys. The angle of incidence 2θ ranged from 20° to 90° at a scanning speed of 2°/min. The microstructure was characterized by optical microscopy (OLYMPUS OLS4000, Tokyo, Japan), scanning electron microscopy (TESCAN VEGA3 LMH, Brno, Czech Republic), electron backscatter diffraction (Zeiss Ultra 55, Oberkochen, Germany), transmission electron microscopy (FEI Tecnai G2 F20, Hillsboro, OR, USA), and high-angle annular dark field scanning transmission electron microscopy (HAADF-STEM). The EBSD data were analyzed using TSL-OIM software. Thin foils 0.2 mm thick for the TEM viewing were punched into thin discs with a 3 mm diameter and mechanically polished, and subsequently subjected to low-angle ion milling using the Gatan precision ion polishing equipment. Bone-shaped tensile specimens were cut from the as-extruded and peak-aged rods with a gauge length of 30 mm and a diameter of 5 mm along the extrusion direction (ED). Using a Shimadzu CMT-5105 (Japan) materials testing machine and an initial strain rate of 0.001 s^−1^, tensile tests were conducted at ambient temperature.

## 3. Results and Discussion

### 3.1. Microstructural Evolution of VWZM10411 Alloy during LDTE Process

**Figure 2:**
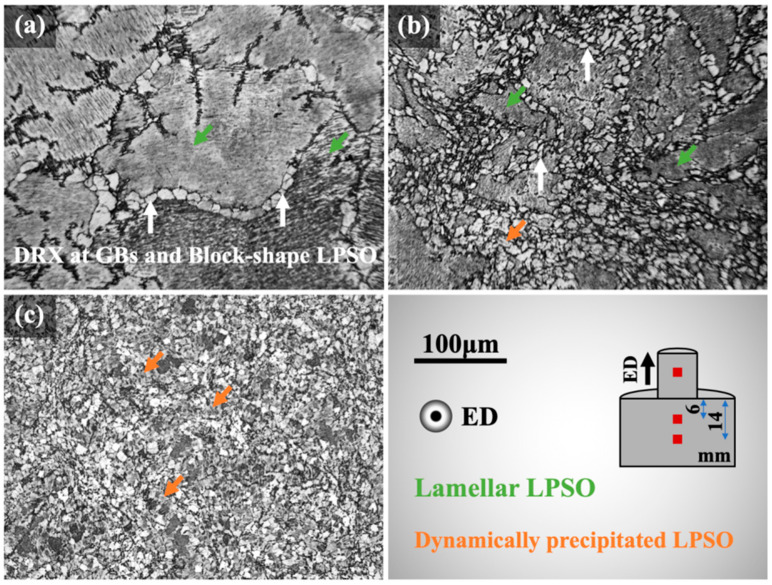
Metallographic micrographs of the partially extruded E2 alloy: (**a**) and (**b**) observed at the locations of 14 and 6 mm before the die entrance; (**c**) as-extruded sample.

The metallographic microstructure of the E2 alloy is shown at various positions prior to the die entrance in the LDTE process in Figure 2. The schematic plot on the bottom right indicates the sampling sites. During the early period of extrusion (Figure 2a), the nucleation and growth of recrystallized grains occurred around the grain boundaries and interphase boundaries between the α-Mg and blocky LPSO (14H, Mg12(Gd, Y)Zn) phases, consistent with the particle-induced recrystallization mechanism [25]. Simultaneously, the laminated LPSO phases within the grains initiated bends and twists. As the LDTE process continued (Figure 2b), more and more recrystallization grains nucleated and grew close to the grain and interphase boundaries, further increasing the recrystallisation volume fraction. Dislocation motion within the torsional zone of some block LPSO phases surpassed their load-bearing capacities, resulting in crushing [26,27]. The pulverized LPSO phases were scattered around the grain boundaries and stimulated recrystallization through the particle-induced mechanism, while also providing a dragging effect on the grain boundaries and impeding the recrystallized grains from coarsening. As the extrusion procedure continued (Figure 2c), some recrystallized grains experienced a dynamic precipitation of laminated LPSO phases, while the laminated LPSO phases within hot-worked grains became progressively more twisted and distorted. The twisted laminated LPSO phases tended to cause stress concentrations between them, leading to a high density of dislocation entanglement until the twisting of the LPSO phase was insufficient to completely discharge the local strain, at which point recrystallization grain nucleation and growth occurred between α-Mg and twisted laminated LPSO phases [28]. In spite of a low-extrusion die temperature (390 °C), the dynamic recrystallization of the VWZM10411 alloy produced by the LDTE process was almost complete on account of the high ingot temperature (510 °C).

### 3.2. Microstructure of as-Extruded VWZM10411 Alloys

**Figure 3:**
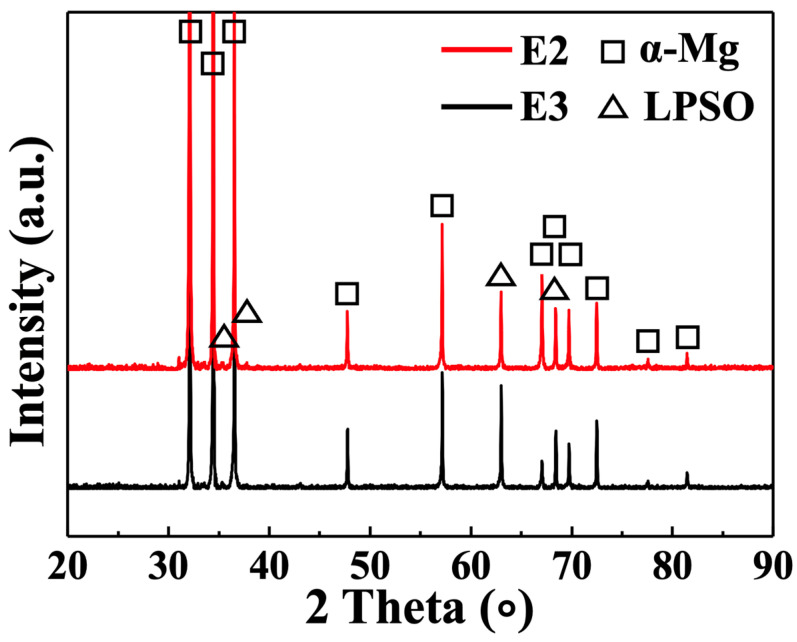
XRD patterns of as-extruded E2 and E3 samples.

The XRD patterns of the as-extruded E2 and E3 alloys are depicted in Figure 3. The main phase compositions of the extruded alloys are α-Mg and 14H-type LPSO phases, according to the research analysis of XRD patterns. This is the same phase composition as Mg-RE-Zn alloys in their heat-treated state [29], as previously reported, demonstrating that the LDTE process does not affect the phase type of the alloy during extrusion. A comparison of the characteristic diffraction peaks of the LPSO phase for the isothermally extruded E3 alloy and those for the LDTE-processed E2 alloy shows that the latter has a slightly higher intensity, indicating a slightly higher content of the LPSO phase in the E2 alloy.

**Figure 4:**
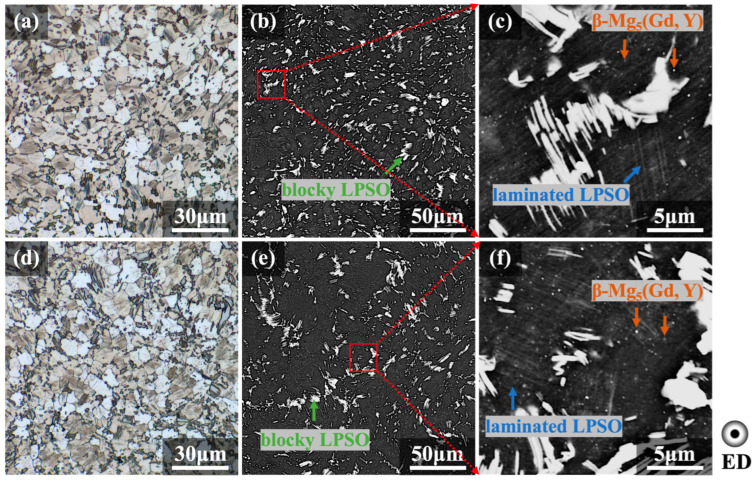
Metallographic and SEM-BSE images of as-extruded alloys observed on transverse sections: (**a**–**c**) E2 and (**d**–**f**) E3 alloys.

**Figure 5:**
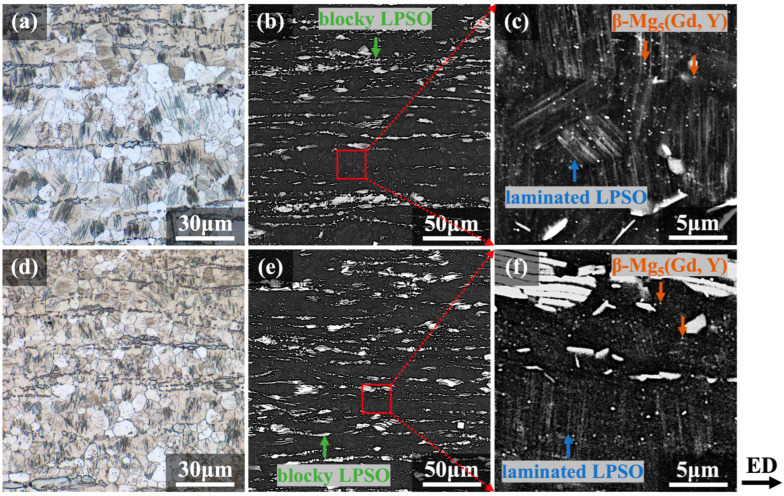
Metallographic and SEM-BSE images of as-extruded alloys observed on longitudinal sections: (**a**–**c**) E2 and (**d**–**f**) E3 alloys.

Figure 4 and Figure 5 show the metallographic and SEM-BSE microstructures of the extruded E2 and E3 alloys in transverse and longitudinal sections, respectively. Both samples underwent a high degree of recrystallization after different hot extrusion processes. The α-Mg grains in the E2 and E3 alloys were mostly distributed equiaxially in both transverse and longitudinal sections, and the average grain sizes of the recrystallized grains were ~7.4 and ~6.1 μm for the two alloys, respectively. During the extrusion process, the laminated LPSO phase was dynamically precipitated and distributed inside the grains parallel to each other, with their orientation related to the grain orientation. The blocky LPSO phase is usually formed after the coarse bulk LPSO phase has broken up in the pre-extrusion heat-treated alloy [30]. The distribution of blocky LPSO phases in the transverse section is diffuse, occurring at the grain boundaries, while in the longitudinal section, it is elongated along the ED and arranged in a streamline pattern. The blocky LPSO phases tend to coordinate the deformation by twisting and folding under extrusion stress [31,32].

As shown in Figure 4c,f and Figure 5c,f, many β-Mg_5_(Gd, Y) phases are diffusely precipitated within the grains and around grain boundaries in the form of fine particles with a bright white contrast. Figure 4c,f and Figure 5c,f are enlarged views from Figure 4b,e and Figure 5b,e, respectively, as indicated by the red boxes. In view of the small size and low quantity in volume of such dynamically precipitated particulates, β-Mg_5_(Gd, Y) phases could not be detected in the metallographic analysis during extrusion in Figure 2 or the XRD analysis in Figure 3. Β-Mg_5_(Gd, Y) particles with favorable thermal stability properties were diffusely distributed, and the component distributed at the grain boundaries could significantly contribute to pinning, which prevented recrystallized grains from becoming coarser during the hot extrusion process [33,34,35]. The dynamic precipitation driving force should be stronger in the LDTE-processed sample on account of lower die temperatures, which cause higher degrees of supersaturation during extrusion. Therefore, a comparison of Figure 4c,f and Figure 5c,f suggests that volumetric proportions of laminated LPSO phases and β phases are slightly higher in the E2 sample than in the E3 sample, and this is in accordance with the results of the XRD analysis shown in Figure 3.

**Figure 6:**
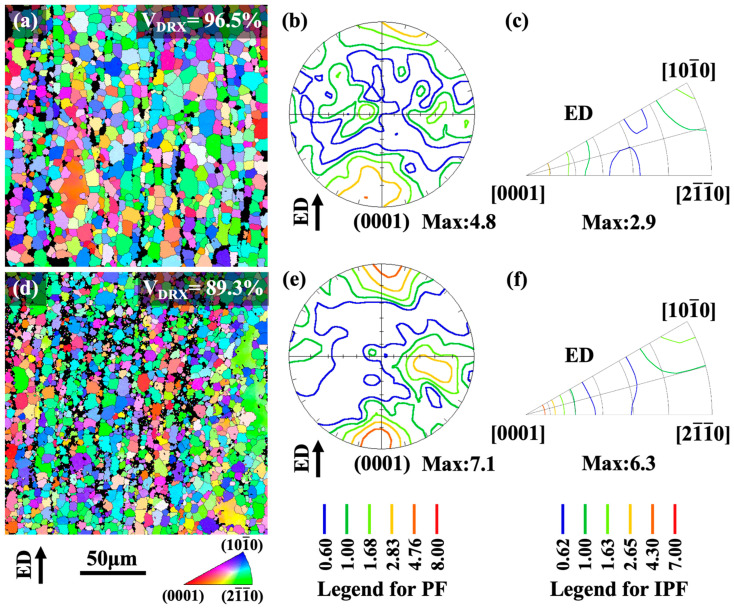
EBSD results of as-extruded E2 (**a**–**c**) and E3 (**d**–**f**) alloys on the longitudinal section: (**a**,**d**) IPF map; (**b**,**e**) (0001) pole figures; (**c**,**f**) inverse pole figures referring to ED.

The EBSD micrographs taken from the lengthwise profiles of the as-extruded E2 and E3 alloys are shown in Figure 6. Defining the recrystallization zone as grains with an intracrystalline orientation difference of less than 2°, it can be statistically observed that sample E2 has a larger average grain size as well as a slightly higher recrystallization volume fraction (96.5%) than that of sample E3 (89.3%). As a result, the peak texture intensity of the former (4.8 MRD) is slightly weaker than that of the latter (7.1 MRD), and the grain orientation is more random. The as-extruded alloy had a double-texture component, as shown by the (0001) pole diagrams and IPF figures, consisting of a typical fiber texture component with a $<10\bar{1}0>$_Mg_ orientation along the ED and an anomalous texture component oriented with (0001) base plane perpendicular to the ED. Previous studies [36,37] attributed the formation of anomalous textural compositions in Mg-RE-Zn alloys to anisotropic changes in the migration kinetics of grain boundaries due to the segregation of RE elements at the grain boundaries. Therefore, the anomalous texture intensity improves with elevated amounts of recrystallization and larger grain sizes. Both the E2 and E3 alloys had a high degree of recrystallization, which resulted in a more intense recrystallization-induced anomalous texture component than the typical fiber texture component. This is in stark contrast to the texture component of the Mg-RE-Zn alloy that features a bimodal microstructure [38]. It is suggested from the experimental data that the E2 alloy has a much higher percentage by volume of recrystallized grains, which results in a more random grain orientation and a weaker texture intensity compared to the E3 alloy. In general, the double-texture component in the extruded alloys comprises a typical fiber texture component and an anomalous texture component, and the high rate of recrystallization causes a stronger anomalous texture component compared to the fiber texture component.

### 3.3. Tensile Properties of as-Extruded VWZM10411 Alloys

**Figure 7:**
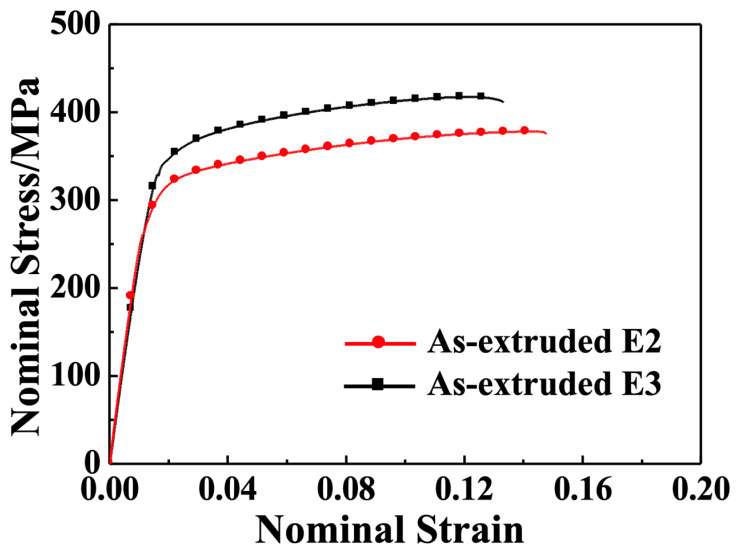
Tensile properties of the as-extruded samples tested along ED at ambient temperature.

The engineering stress–strain diagram of the as-extruded E2 and E3 alloys at room temperature is plotted in Figure 7, and the associated mechanical properties are tabulated in Table 2. The data indicate that the isothermally extruded E3 alloy displays a superior tensile strength with a UTS of 417 MPa, TYS of 336 MPa, and elongation of 11.5%. Even though the LDTE-processed E2 alloy did not have as strong a tensile strength as the E3 alloy, its extrusion rate was almost 6.7-times faster than the E3 alloy. Meanwhile, the E2 also showed good overall mechanical properties, with a UTS of 378 MPa, TYS of 298 MPa, and elongation of 13.1%. This suggests that the E2 alloy can be used in certain applications that require a relatively high extrusion rate and favorable mechanical properties.

On the basis of the microstructural observation, the strengthening mechanism was primarily related to solid-solution strengthening (${∆\sigma }_{\mathrm{solution}}$), grain boundary strengthening ($∆\sigma_{\mathrm{GB}}$), and other strengthening (${∆\sigma }_{\mathrm{other}}$), such as texture strengthening, fiber strengthening, dispersion strengthening, et cetera. Tensile yield strength (${∆\sigma }_{\mathrm{TYS}}$) can therefore be expressed as:

(1) $$\sigma_{\mathrm{TYS}}=\sigma_{0}+{∆\sigma }_{\mathrm{solution}}+∆\sigma_{\mathrm{GB}}+{∆\sigma }_{\mathrm{other}}$$

The frictional force of the dislocation sliding on the slip ($\sigma_{0}$) in the Mg-Gd system alloys was assumed to be 46 MPa [14,39].

For the solid-solution strengthening effect in the extruded E2 and E3 alloys, with a composition of VWZM10411 (converted to an atomic ratio of Mg-1.83Gd-1.38Y-0.38Zn-0.36Mn (at.%)), the calculated solubilities of Gd and Y in the equilibrium solid state of the α-Mg matrix were ∼1.56% and ∼1.24% at 470 °C, correspondingly, according to the thermodynamic database of the Pan-Mg 2017 version [40]. As a consequence of the rapid cooling rate after extrusion, the α-Mg matrix was in an oversaturated solid-solution state. Here, Gd and Y play an essential role in solid-solution strengthening, which can be determined by the equation below [41]:

(2) $${∆\sigma }_{\mathrm{solution}}=\sigma_{\mathrm{solution}}-\sigma_{\mathrm{pure}}={\left(\sum_{i}k_{i}^{1/n}c_{i}\right)}^{n}={\left(k_{\mathrm{Gd}}^{1/n}c_{\mathrm{Gd}}+k_{Y}^{1/n}c_{Y}\right)}^{n}\;$$

The solid-solution yield strength ($\sigma_{\mathrm{solution}}$) and pure Mg yield strength ($\sigma_{\mathrm{pure}}$) are important parameters for understanding the mechanical behavior of materials. The strengthening constant for component i in the solid solution (k_i_) and the concentration of component i in the solid solution (c_i_) can be used to calculate the strengthening effect of the solid solution in magnesium alloys. The constant n is equal to 1/2. The values of the strengthening constants for Gd (k_Gd_) and Y (k_Y_) are 683 and 737 MPa, correspondingly.

The proportions of Mg_12_(Gd, Y)Zn phases by volume in the E2 and E3 alloys were approximately 11.1% and 13.8%, respectively. The proportions of Mg_5_(Gd, Y) phases by volume in the E2 and E3 alloys were approximately 0.9% and 1.2%, respectively. By combining the atomic ratios for each element and the proportions by volume of the phases, the concentrations of Gd and Y in the α-Mg matrix could be estimated to be approximately 1.47% and 1.11%, respectively, in the E2 alloy and 1.36% and 1.02%, respectively, in the E3 alloy. These concentrations remained below the theoretical degree of solid-solution equilibrium for Gd/Y elements in the Mg phase at a high temperature, which allowed for the complete dissolution of these atoms in the Mg phase, thus promoting solid-solution strengthening. The strengthened effects of the solid solution in the E2 and E3 alloys could be calculated to be ${∆\sigma }_{\mathrm{solution}-2}$ ≈ 114 MPa and ${∆\sigma }_{\mathrm{solution}-3}$ ≈ 109 MPa, respectively.

For grain boundary strengthening, the average grain sizes of the E2 and E3 alloys with a high level of recrystallization were approximately 7.4 and 6.1 μm, correspondingly. The grain boundary strengthening values for the extruded E2 and E3 alloys were approximately 60 and 66 MPa, respectively, according to the Hall–Petch relationship ($∆\sigma_{\mathrm{GB}-2}$ ≈ 60 MPa and $\sigma_{\mathrm{GB}-3}$ ≈ 66 MPa).

Since the yield strengths of the as-extruded E2 and E3 alloys were 298 and 336 MPa, respectively, the other strengthening effects could be calculated as ${∆\sigma }_{\mathrm{other}-2}=\sigma_{\mathrm{TYS}-2}{-\;\sigma }_{0}-{∆\sigma }_{\mathrm{solution}-2}-∆\sigma_{\mathrm{GB}-2}$≈ 298 MPa − 46 MPa − 114 MPa − 60 MPa = 78 MPa and ${∆\sigma }_{\mathrm{other}-3}=\sigma_{\mathrm{TYS}-3}{-\sigma }_{0}-{∆\sigma }_{\mathrm{solution}-3}-∆\sigma_{\mathrm{GB}-3}$ ≈ 336 MPa − 46 MPa − 109 MPa − 66 MPa = 115 MPa.

**Figure 8:**
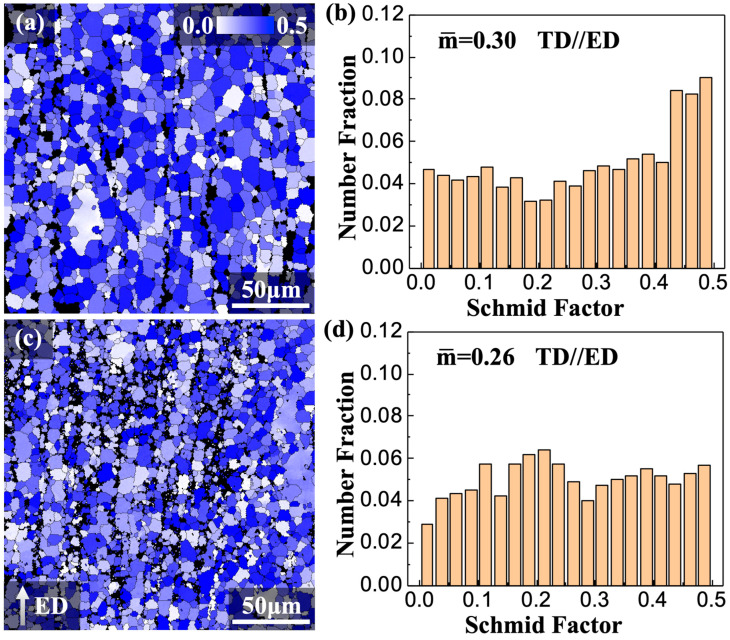
Schmid factor distribution maps and histograms of the as-extruded alloys when the tensile stress is applied along the ED: (**a**,**b**) E2 and (**c**,**d**) E3 alloys.

The effect of texture strengthening, reflected in the variation in the Schmidt factor distribution state, also played a significant role in material strengthening. To gain further insights, the Schmidt factor distributions of the (0001)$<11\bar{2}0>$ basal slip, the principal deformation mechanism in extruded the E2 and E3 alloys, were calculated and are presented in Figure 8. The results show that when the direction of tension is arranged along the ED, the mean Schmidt factors of the E2 and E3 alloys are 0.30 and 0.26, respectively. This suggests that (0001)$<11\bar{2}0>$ slipping is more difficult for the dislocations within the E3 alloy in comparison to the E2 alloy. The relationship between the Schmidt factors for basal slips and the recrystallization degree and texture characteristics of the alloy in ED was well established [36,42]. In the hot-worked grain, the Schmidt factor was close to 0 and could be considered negligible. As a result, alloys with lower recrystallization degrees tend to have lower average Schmidt factors. However, both the E2 and E3 alloys exhibited a significant anomalous texture, with a large angular deviation between the <0001> direction of most magnesium grains and the ED. This led to a more uniform distribution of different Schmidt factors in the histogram.

In addition, the average lengths of blocky LPSO phases measured lengthwise to the ED in the E2 and E3 alloys were about 20 and 14 μm, respectively, with the average spacing values perpendicular to the ED being about 10 and 8 μm, respectively. The magnitude and spacing of blocky LPSO phases were too large to allow them to directly interact with dislocations and promote strengthening [43]. Instead, short fiber reinforcement was primarily generated by the blocky LPSO phase [44,45]. The E3 alloy exhibited a smaller blocky LPSO phase and a more diffuse Mg_5_(Gd, Y) phase, resulting in stronger strengthening (${∆\sigma }_{\mathrm{other}}$) compared to the E2 alloy by ~37 MPa. The lamellar LPSO phase together with the diffuse Mg_5_(Gd, Y) phase mainly contributed to diffuse reinforcement.

### 3.4. Microstructure and Tensile Properties of Peak-Aged VWZM10411 Alloys

**Figure 9:**
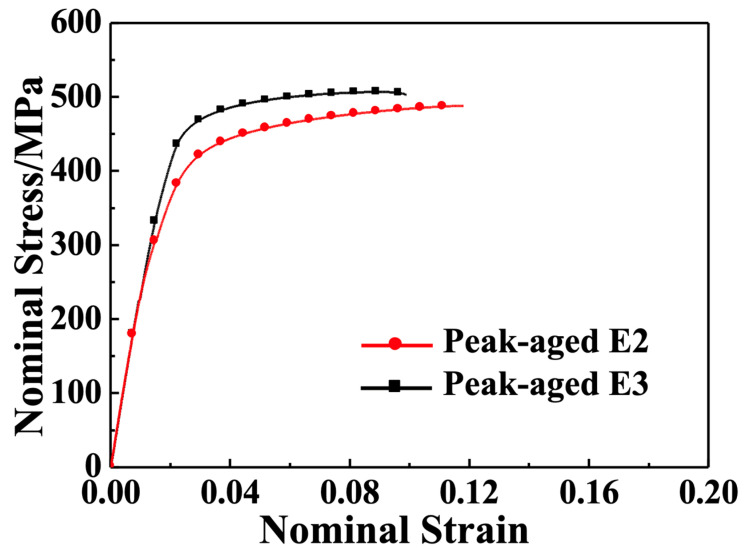
Tensile properties of the peak-aged E2 and E3 samples tested along the ED at ambient temperature.

Figure 9 depicts the engineering stress–strain curves of the E2 and E3 alloys along the ED after 50 h of aging at 200 °C. Table 2 also summarizes the mechanical property data of peak-aged alloys. In general, treatment with isothermal aging results in a considerable enhancement of the strength and a decline in the plasticity of both the E2 and E3 alloys. The UTS of the E2 alloy increased by 110 MPa to reach 488 MPa, while the TYS of the E2 alloy increased by 46 MPa to reach 324 MPa. However, its elongation decreased by 3.4% to 9.7%. The E3 alloy also exhibited an improvement in strength, with the UTS increasing by 90 MPa to 507 MPa, TYS increasing by 83 MPa to 419 MPa, and elongation decreasing by 3.8% to 7.7%.

The Mg-Gd-Y-Zn alloys displayed impressive thermal stability when subjected to isothermal aging at 200 °C. The grain size, recrystallization state, distribution of second-phase states at the micrometer scale, and texture characteristics closely resembled those observed in the extruded state [46]. This suggests that the differences in strengthening mechanisms between the aged samples and the extruded state are primarily attributed to solid-solution strengthening and precipitation strengthening, as indicated by the following equation:

(3) $$\sigma_{\mathrm{TYS}-A}{=\sigma }_{0}+{{∆\sigma }_{\mathrm{solution}-A}+{∆\tau }_{\mathrm{PS}-A}+∆\sigma }_{\mathrm{GB}}+{∆\sigma }_{\mathrm{other}}$$

A symbolizes the aged-state sample. ∆τ_PS_ symbolizes the precipitation strengthening effect. At 200 °C, the balanced solubilities of Gd/Y elements in the Mg matrix are similar (~0.61 at.%), and concentrations of Gd/Y solid-solution elements in the Mg matrix are almost equal in peak-aged E2 and E3 alloys, indicating that their solid-solution strengthening effect is relatively similar. Equation (2) can be used to estimate their solid-solution strengthening effect, ∆σ_solution-A_ ≈ 78 MPa. With reference to Equation (3), the precipitation strengthening effect experimentally measured in the E2 and E3 alloys can be calculated as follows, respectively:

$${∆\tau }_{\mathrm{PS}-2A}=\sigma_{\mathrm{TYS}-2A}-\sigma_{0}-\;{∆\sigma }_{\mathrm{solution}-A}-∆\sigma_{\mathrm{GB}-2}-{∆\sigma }_{\mathrm{other}-2}=82\;\mathrm{MPa}$$

$${∆\tau }_{\mathrm{PS}-3A}=\sigma_{\mathrm{TYS}-3A}-\sigma_{0}-{∆\sigma }_{\mathrm{solution}-A}-∆\sigma_{\mathrm{GB}-3}-{∆\sigma }_{\mathrm{other}-3}=114\;\mathrm{MPa}$$

**Figure 10:**
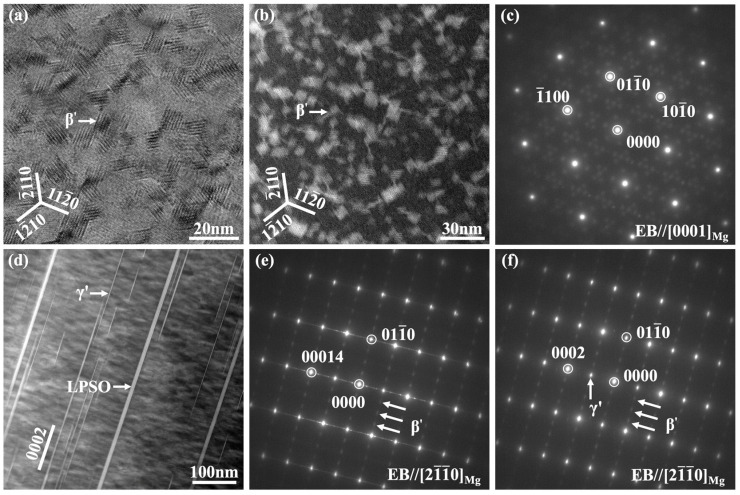
TEM micrographs of peak-aged E2 sample and corresponding SAED patterns: (**a**) HRTEM, (**b**) HAADF-STEM, and (**c**) SAED patterns with EB//<0001>_Mg_; (**d**) HAADF-STEM and (**e**,**f**) SAED patterns with EB//<$2\bar{1}\bar{1}0$>_Mg_.

To verify the consistency between the experimental values of the precipitation strengthening effect, ∆τ_PS_, obtained from the calculations and the theoretical values calculated by the Orowan model, Figure 10 shows a view of the TEM microstructure obtained from the peak-aged E2 sample and the associated diffraction spot patterns that were selected for the analysis. The electron beam directions are EB//<0001>_Mg_ in Figure 10a–c, and EB//$<2\bar{1}\bar{1}0>$_Mg_ in Figure 10d–f. The predominant precipitated strengthening phases were prismatic β′ and basal γ′ phases, with a limited number of chain-like β″ phases distributed between the β′ phases [47,48]. The β′ phases exhibited a close size distribution in the $<2\bar{1}\bar{1}0>$_Mg_ and $<10\bar{1}0>$_Mg_ directions, with a size of around 10 nm. The β′-phase size in the <0001>_Mg_ direction was approximately 45 nm. As the thickness of the γ′ phase was constant and equal to a single-layer dislocation, it was not visible at EB//<0001>_Mg_. The γ′ phase length in the $<10\bar{1}0>$_Mg_ direction varied from few tens of nanometers to a few micrometers. To simplify the calculation, it was assumed that the primary mechanism of deformation was governed by basal slips and that the precipitates were circumvented by the dislocations. Since the basal precipitated phase has a less significant effect on hindering the basal slip compared to the prismatic precipitated phase, it is feasible to calculate the Orowan strengthening mechanism using the following equation [49,50,51]:

(4) $$∆\tau_{\mathrm{PS}-O}=\frac{\mathrm{Gb}}{2\pi \sqrt{1-\;v}}\frac{1}{\lambda }\ln\frac{d_{p}}{r}$$

where G is the shear modulus (~16.6 GPa), d_P_ is the average diameter of the precipitates on the sliding plane (~10 nm), b is the Burgers vector (~0.32 nm), v is the Poisson’s ratio (~0.3), λ is the equivalent spacing of precipitates on the slip plane (~40 nm), and r is the radius of the dislocation nucleus (~0.32 nm) [51]. The calculation results show that the peak-aged E2 alloy exhibits a precipitation strengthening effect of ~87 MPa according to the Orowan model, which is relatively close to the experimental value of ∆τ_PS_ (~82 MPa).

**Figure 11:**
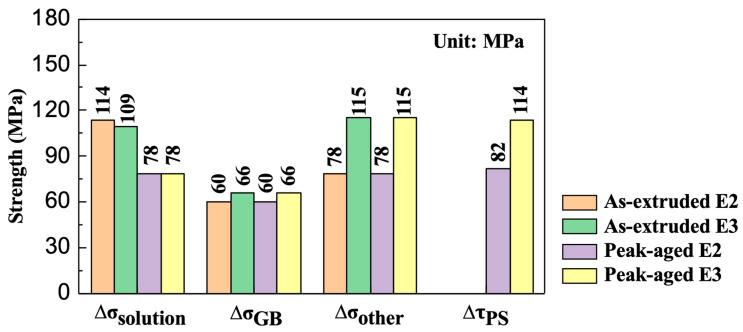
Quantitative statistics of various strengthening mechanisms in as-extruded and peak-aged E2 and E3 alloys.

To better illustrate the quantitative statistical results of the individual strengthening mechanisms in the E2 and E3 alloys in their as-extruded and peak-aged states, an overview of the findings from the above mentioned analysis is summarized in Figure 11 and Table 3. Through a comprehensive analysis and investigation, it was demonstrated that the strength of the as-extruded E2 and E3 alloys as well as the peak-aged E2 and E3 alloys was substantially influenced by various strengthening mechanisms, including, but not limited to, solid-solution strengthening, grain boundary strengthening, and precipitation strengthening. The concerted effect of these mechanisms is noteworthy in the enhancement of the alloy’s mechanical performance. Notably, the LDTE technique was initially intended to significantly increase the extrusion speed while lowering the extrusion temperature and increasing the content of solid-solution elements in the extruded alloy, thereby encouraging aging precipitation and enhancing the precipitation strengthening effect. However, the precipitation strengthening of the E2 alloy at a peak age was ~32 MPa less than that of the E3 alloy at its peak age. This might have been due to the fact that the LDTE-processed alloy had a tendency to dynamically precipitate more laminated LPSO and β phases during extrusion, resulting in an alloy with a relatively reduced number of solid dissolved elements (as discussed in Section 3.2). It is known that the presence of the laminated LPSO phase in Mg-RE-Zn alloys significantly enhances the critical resolved shear stress for basal slip, promoting non-basal slip activation. This enhancement in strength, coupled with the effective reduction in internal stresses within grains and along the grain boundaries serves as a preventive measure against microcrack initiation and ultimately enhances the alloy’s toughness [52].

## 4. Conclusions

The impact of extrusion process parameters on the microstructure and mechanical properties of the high-strength Mg-10.3Gd-4.4Y-0.9Zn-0.7Mn alloy was systematically studied in this study. It was demonstrated that the large differential-thermal extrusion process was a highly effective method for increasing the extrusion speed as well as refining the microstructure of these alloys. This technique enabled the effective preparation of high-strength magnesium alloys with good surface quality and superior mechanical properties. The following conclusions can be drawn:

(1) The phase composition of the high-strength VWZM10411 alloy produced via the LDTE process is consistent with that of conventionally hot extruded alloys. The primary phases present in the LDTE-processed alloy are α-Mg, 14H-type LPSO, and Mg_5_RE. During extrusion, the LPSO and Mg_5_RE phases are crucial in preventing recrystallized grains from becoming coarser, and the LPSO phase is significant in coordinating the deformation via twisting and bending.

(2) The LDTE-processed alloy possesses an average grain size of ~7.4 μm, which is slightly coarser compared to the isothermal extruded alloy (~6.1 μm). With a texture that includes both the <0001>_Mg_//ED anomalous texture component and $<10\bar{1}0>$_Mg_//ED fiber texture component, the preferred orientation of the LDTE-processed alloy is weaker. The texture intensity of the LDTE-processed alloys is also lower, with a value of 4.8 MRD compared to 7.1 MRD for normal isothermal extrusion.

(3) After isothermal aging at 200 °C, the LDTE-processed sample with a UTS of 488 MPa, TYS of 344 MPa, and elongation of 9.7% exhibits lower strength properties but higher ductility than the isothermally extruded sample with a UTS of 507 MPa, TYS of 419 MPa, and elongation of 7.7%, which do not detract from the fact that the LDTE is a promising process for the efficient preparation of high-strength Mg alloys. The enhancement of strength of the age-hardened sample is considered to be caused by a combination of various strengthening mechanisms, which comprise solid-solution strengthening, grain boundary strengthening, precipitation strengthening, and other strengthening (including fiber strengthening, texture strengthening, dispersion strengthening, etc.). The quantitative contributions of these mechanisms to the TYS are determined to be ~78, ~60, ~82, and ~78 MPa, respectively.

## Figures and Tables

**Table 1:** Extrusion processes with various extrusion parameters.

Designation	Thermal Condition			
	Ingot Temperature (°C)	Die Temperature (°C)	Ram Speed (mm/s)	Extrusion Ratio
E1	510	390	1.5	11:1
E2	510	390	2.0	11:1
E3	450	450	0.3	11:1
E4	450	450	2.0	11:1
E5	510	450	0.3	11:1
E6	510	450	2.0	11:1

**Table 2:** Tensile properties of as-extruded and peak-aged alloys at ambient temperature.

Alloys	UTS (MPa)	TYS (MPa)	EL (%)
As-extruded E2 sample	378	298	13.1
As-extruded E3 sample	417	336	11.5
Peak-aged E2 sample	488	344	9.7
Peak-aged E3 sample	507	419	7.7

**Table 3:** Quantitative statistics of various strengthening mechanisms in as-extruded and peak-aged E2 and E3 alloys.

Alloys	${∆\sigma }_{\mathrm{solution}}$	$∆\sigma_{\mathrm{GB}}$	${∆\sigma }_{\mathrm{other}}$	∆τ_PS_
As-extruded E2 sample	114	60	78	-
As-extruded E3 sample	109	66	115	-
Peak-aged E2 sample	78	60	78	82
Peak-aged E3 sample	78	66	115	114

Note: other strengthening, ${∆\sigma }_{\mathrm{other}}$, encompasses additional reinforcement mechanisms that are challenging to quantify, such as texture strengthening, fiber reinforcement, and dispersion strengthening.

## Data Availability

The raw/processed data required to reproduce these findings cannot be shared at this time as the data also form part of an ongoing study.
